# Moving beyond the empty cell: The threat of decontextualized healthcare data

**DOI:** 10.1371/journal.pdig.0001194

**Published:** 2026-01-13

**Authors:** Aya El Mir, Eric Bezerra de Sousa, Ignacio Mesina-Estarrón, Leo Anthony Celi, Moad Hani, Mohammed Benjelloun, Neha Nageswaran, Saïd Mahmoudi, Shaheen Siddiqui, Sreeram Sadasivam, William Greig Mitchell

**Affiliations:** 1 Department of Engineering, New York University Abu Dhabi, Abu Dhabi, United Arab Emirates; 2 Faculty of Medicine, University of Sao Paulo, Sao Paulo, Brazil; 3 Department of Neurosurgery, Children’s Hospital of Orange County, Orange, California, United States of America; 4 Laboratory for Computational Physiology, Massachusetts Institute of Technology, Massachusetts, United States of America; 5 Division of Pulmonary, Critical Care and Sleep Medicine, Beth Israel Deaconess Medical Center, Boston, Massachusetts, United States of America; 6 Department of Biostatistics, Harvard T.H. Chan School of Public Health, Boston, Massachusetts, United States of America; 7 Department of Computer Engineering and Management, Faculty of Engineering, University of Mons (UMONS), Mons, Belgium; 8 Robotic Assistant Systems, Carl von Ossietzky Universität Oldenburg, Oldenburg, Germany; 9 GeneSilico, Inc., Austin, Texas, United States of America; 10 Software Engineering, EGYM, Munich, Germany; 11 Department of Ophthalmology, Cambridge University Hospitals, Cambridge, United Kingdom; National Tsing-Hua University: National Tsing Hua University, TAIWAN

## Abstract

Missing, inaccurate, or poorly documented data in healthcare is often treated as a technical problem to be statistically resolved via imputation, deletion, or modeling assumptions about randomness. However, such inaccuracies relate to far more complex socioeconomic and geopolitical issues, rather than “errors of data entry” to be ameliorated with statistical modeling techniques. We outline that what is really missing or inaccurate is the *context* in which the data is collected—and that only by understanding this context can we begin to prevent artificial intelligence’s (AIs) amplification of misleading, decontextualized data. We critically examine how traditional modeling methods fail to account for the factors that influence what data gets recorded, and for whom. We show how AI systems trained on decontextualized data reinforce health inequities at scale. And, we review recent literature on context-aware approaches to understanding data, that incorporate metadata, social determinants of health, fairness constraints, and participatory governance to build more ethical and representative systems. Our analysis urges the AI and healthcare communities to move beyond the traditional emphasis on statistical convenience, toward socially grounded and interdisciplinary strategies for handling decontextualized data.

## Author summary

Healthcare data that is missing, incomplete, or inaccurately documented is often treated as a technical problem to be solved with statistical methods. We emphasize that this perspective overlooks the real issue: the data has been stripped of its context. Missing, incomplete, or inaccurate data (collectively termed *decontextualized data*) is not random; it is shaped by human decisions, social barriers, and systemic inequalities. Decontextualized healthcare data becomes increasingly dangerous as the use of AI in healthcare proliferates. Models trained on decontextualized data learn existing distortions as if objective truths. Consequently, their predictions risk reinforcing the very inequities that caused the flawed data in the first place and exacerbating health disparities at scale. We argue for a paradigm shift towards understanding why data becomes decontextualized. This requires a concerted effort between machine learning communities and domain experts who understand data context. It is only through this partnership that we can begin to build models that account for the complex realities embedded in decontextualized healthcare data that cannot be solved by sophisticated modeling techniques alone.

## 1. Introduction

Statistical techniques to manage missing, inaccurate, or poorly documented healthcare data like imputation and deletion have been extensively researched and applied for decades, on the premise that data is missing either completely at random (MCAR), at random (MAR), or not at random (MNAR) [1,2]. These techniques, however, ignore an incontrovertible truth about such healthcare data: that it is essentially *never* missing, inaccurate, or flawed at random. What is really missing is an understanding of the *context* of the data. Rather than representing a data cell to be imputed, deleted, or filled for better statistical model performance, such data is the result of long-standing, intertwining geopolitical and socio-economic complexities, which demand far more nuanced and insightful approaches than those currently used if we are to meaningfully interpret them.

When approaching missing, inaccurate or poorly documented healthcare data, statistical methods rarely appreciate how the data came about—how data was collected (or not collected) and by whom, the socio-economic policies or habits facilitating data collection (or not), the devices used to capture certain data signals (or mis-capture/not capture signals), and how final datasets are curated and made available for analysis [3].

Data may be missing because of socioeconomic barriers to documentation. For instance, in intensive care units (ICU), overnight blood sugar level (BSL) data may not be documented due to language discordance between staff and patient, necessitating an overnight interpreter and extra work—it’s easier to let the non-English speaking patient sleep without checking their BSL, rather than wake them and concurrently find a translator [4]. Similarly, overnight bed-ulcer status might not be documented because a patient is too obese to roll with skeleton night staff, so it’s decided to wait till the morning. These data aren’t just “missing;” they’re the result of pernicious social barriers prohibiting data collection.

Data may not make it to the point of collection in the first place—a form of “invisible” missing data. Black patients suffering out-of-hospital cardiac arrest (OOHCA) are less likely to be resuscitated and subsequently transported to a medical facility [5], making their data less likely to be captured. Aboriginal & Torres Strait Islander Australians (hereafter “Indigenous Australians”) are more likely to self-discharge from ICU against medical advice, similarly making their critical illness data impossible to capture in the first place [6]. And female patients are known to be more likely to have acute coronary syndrome (ACS) misdiagnosed by male physicians [7], making subsequent cardiovascular data less likely to be recorded.

Even when documented, data may be so inaccurate that the real underlying information is cloaked by erroneous readings. For instance, pulse oximetry data is much more inaccurate for patients with heavily pigmented skin [8], with falsely-high readings leading to systemic under-treatment and worse outcomes [9]. Again, missing data context and subsequent inaccuracy here is not random, but indicative of inequalities in who equipment calibration and data collection has been optimized for (non-pigmented patients) versus who data is being collected from, in a clinical setting.

When AI models are built with decontextualized data, they absorb these blind spots to generate flawed predictions at scale: the well-described “garbage in, garbage out” AI phenomenon [10]. While statistical methods that more accurately address contextual complexities embedded in data may exist, they cannot be developed by those who do not understand the data context at all points in the pipeline, from collection, documentation, curation, storage, and distribution [3]. Current statistical modeling methods to handle missing, inaccurate, and poorly recorded data (hereafter collectively termed “decontextualized data”) dismiss the context dictating why it is flawed and seek only to improve model accuracy parameters. Machine Learning communities working in isolation lack an understanding of the geopolitical and socio-economic context of such missing data and are unable to develop such statistical tools alone. Without this understanding, gaping contextual cavities in data will continue to exist, posing most risk to those who’s data is most contextually-flawed—historically those already disadvantaged by current healthcare structures [11].

This paper is a call to collaborative action between those who understand data context and those developing statistical techniques to resolve decontextualized data. We propose solutions and methodological improvements for tackling decontextualized data so that we might stand a chance at developing insightful methods to minimize its impact on already marginalized populations.

## 2. Rethinking missing data: The importance of context and critique of traditional approaches

Understanding mechanisms that perpetuate decontextualized data requires examining the structural factors underlying data collection. The data lifecycle can be divided into different pipeline stages, to the point of acquisition and use by statisticians, to help highlight geopolitical and socio-economical contextual issues.

### a. Data availability: Historically disadvantaged populations are less likely to have their data collected—“Invisible” missingness contextualization

Healthcare data may be unavailable to document in the first place, a form of ‘invisible’ missing data. This data is traditionally representative of patients from lower socioeconomic backgrounds, racial or ethnic minority groups, or communities with fragmented healthcare services across multiple regions. These are the populations whose healthcare stands most to gain by the considered use of AI, but who will conversely become relatively more disadvantaged without adequate contextual understanding of their healthcare data.

The health-related disadvantage of Indigenous Australians is extensively described, with substantially higher rates of chronic disease and difficulty accessing primary or tertiary healthcare [12–16]. Self-discharge rates against medical advice for Indigenous Australians are the highest in the world [17]; indeed, they’re almost four-times more likely to self-discharge during an ICU admission than non-Indigenous Australians [6]. Whilst the reasons for self-discharge are complex, they likely reflect an unfavorable perception of the unfamiliar hospital environment, perceived differences in care, institutionalized racism, and/or communication barriers [6,18,19]. Those who self-discharge against medical advice have almost 50% higher 8-year mortality compared to those who do not self-discharge against medical advice (adjusted hazard ratio 1.46; 95% confidence interval 1.01–2.1) [6]. Despite being a particularly high-risk group, healthcare data for patients who self-discharge against medical advice becomes impossible to document, and its omission from morbidity/mortality prediction models means actual predictions underestimate how unwell these patients are.

“Invisible” missing data similarly applies to Black patients suffering OOHCA in the United States (US). Black patients experience significantly higher rates of OOHCA than White patients [20] and in rural US settings are up to five times less likely to survive OOHCA compared to patients living in more affluent areas of the country [21]. Survival discrepancies following OOHCA are *not* due to biological variation between Black vs White patients—but contextual factors: lower likelihood of OOHCA being witnessed by bystanders, lower likelihood of bystander cardiopulmonary resuscitation if witnessed, lower availability of automatic electronic defibrillators, longer Emergency Response Service times and lower likelihood of early advanced airway management upon their arrival [5]; leading to lower rates of hospitalization after OOHCA. Further, those that do make it to hospital are less likely to receive urgent interventions correlated with survival, like targeted temperature management, coronary angiogram, or cardiac catheterization due to variability in hospital capabilities (i.e., 24/7 cardiac catheter labs) and standardized post-arrest pathways [5,21]—further contextualizing why survival rates are poorer. Decontextualized data used to train models to predict survival after OOHCA for Black patients omit the data for those who did not make it in the first place, and for those who do, may erroneously predict substantially higher risk of mortality attributed toward their race, rather than the context of where data was collected, i.e., in lower capability healthcare settings. Without an understanding of this context, lifesaving interventions may be withdrawn earlier on the assumption that their outcome will be poor due to their race.

When certain populations are systematically underrepresented in datasets, such as Indigenous Australians or Black patients in the US, predictive models can also underestimate future healthcare needs. By failing to understand the data context, models misinterpret that relatively lower historical healthcare expenditure (which is actually due to relatively under-reported healthcare data) equates to lower future healthcare needs than are actually required—as has been demonstrated by Obermeyer et al [22].

### b. Data collection and documentation: Incomplete, inaccurate, or misleading documentation due to clinician discretion or workforce variability—Clinical environment contextualization

Even when recorded, healthcare data is often incomplete, inconsistent or misleading due to contextual conditions under which measurements are recorded. For example, the frequency of BSL measurements in ICU varies between patients depending on physician judgement, anxiety, and perceived risk of hypoglycemia. When patients are perceived by clinicians as more at risk of hypoglycemia, possibly due to clinician anxiety or perceived (rather than actual) risk, they are more likely to have more frequent BSL measurements. A recent review of the MIMIC-IV dataset analyzing almost 25,000 patients suggested as much, demonstrating that Black and Hispanic patients underwent BSL measurements 6% and 11% more frequently than White patients respectively, even when fully adjusted for illness severity and comorbidities (95% incidence rate ratio confidence intervals 1.01–1.12, and 1.01–1.21, respectively) [23]. When context is ignored, spurious conclusions may be drawn, i.e., that frequent BSL monitoring is linked to higher illness severity and poorer outcomes, when in reality the reverse causal direction is true, and may actually be more reflective of perceived risk of illness severity and BSL fluctuation, or clinician anxiety [23].

Staffing and shift models in ICU also affect documentation. Workflow interruptions (i.e., staff handover and shift changes) [23] and language barriers (i.e., language discordance between nursing staff and patients) make BSL recording less likely during those periods [4]. The same publication using MIMIC IV data demonstrated that English-speaking patients are 8% more likely to have BSL measurements taken in ICU’s in the US vs non-English speaking patients (95% incidence rate ratio confidence intervals 1.01–1.15), possibly because they’re more likely to “speak up” without language discordance barriers [23]. When such decontextualized data is analyzed, conclusions that certain patients had more frequent readings due to higher illness severity (rather than language concordance) are likely to be drawn. Furthermore, higher ICU mortality for non-English speaking patients without interpreters present is well-documented, independent of BSL measurement frequency [24,25]. Considering less frequent BSL monitoring (which disproportionately affects non-English speaking patients) is also associated with higher BSL fluctuations, higher hospital morbidity/mortality, and longer length of admission [26–29], this is additionally concerning.

### c. Data saving and storage: Understanding the context behind the capacity for consistent, comprehensive data storage

Understanding the context of variation in data storage capacities between healthcare facilities is equally critical. The availability of extensive datasets and significant computational power is an essential precursor to building robust, equitable models [11]. Regional and institutional disparity means those with advanced technological infrastructure and large-scale data storage capabilities, such as affluent regions of the US (home to over 40% of the world’s databases alone), are overrepresented in the data used to train predictive models [11]. Consequently, while models may perform well in environments where data originated, like the US, they lack generalizability and could perpetuate healthcare inequities when applied to populations from data-poor regions, like the Global South [30]. Even within affluent, data-rich areas with large-scale data storage capacity like the US, socially, ethnically, or racially marginalized groups are more likely to have fragmented care across multiple institutions, and lower internet access/literacy preventing the use of online portals and patient-reported outcomes [31]. This worsens scattered, incomplete data documentation for already-marginalized groups within affluent regions, similarly making model findings less generalizable to them. Ignoring the context of data provenance, and limitations imposed by heterogeneous data storage capacities poses further risk to model bias.

### d. How current statistical approaches fail to recognize healthcare data context

Most machine learning pipelines continue to rely on traditional statistical approaches focused solely on data completion. Techniques such as mean, median, and mode imputation; k-nearest neighbors (KNN); regression-based methods; forward and backward filling; interpolation; multiple imputation (including multiple imputation by chained equations (MICE)); and model-based techniques like Kalman filters and mixed-effects models remain widespread [32–34]. These methods, while mathematically sound, fall short in addressing contextual truths embedded in data. They typically assume that missingness can be explained within the data itself, often under the MAR or MCAR paradigms, assumptions that are not only overly optimistic but frequently invalid, ignoring the interplay of socioeconomic and geopolitical factors influencing missing data as described above.

For example, KNN and regression imputation assume similarity or linearity, which fails in marginalized or underrepresented populations where data is systematically lacking. Techniques like multiple imputation may improve model robustness, but their computational cost can increase with data scale, the number of imputations, and imputation model complexity. Crucially, they still rely on the flawed assumption that missingness is ignorable when properly modeled. These approaches prioritize statistical completeness over epistemological clarity, and risk creating an illusion of data integrity while masking the very structural barriers that created the missingness in the first place. Without this awareness of the importance of data context, algorithmic outputs risk reproducing the very inequities they seek to mitigate.

### e. Epistemology: Acknowledging our own limitations

Recognizing missing context requires epistemic humility: acknowledgment that our understanding of data is shaped and limited by our own individual experiences and exposures. Data analysis often proceeds as though those interpreting the data possess full knowledge of its origins. Before any exploratory analysis begins, those responsible for data analysis and modeling, i.e., the machine learning community must ask “how did this data come about?”; acknowledging that the social and structural conditions surrounding data generation are as important as the data itself, to avoid amplifying its embedded biases and inequalities.

## 3. From technical fixes to systemic transformation: Reconceptualizing the AI lifecycle

Logistical decisions and statistical methods applied at early stages of the AI pipeline (i.e., imputation and other downstream adjustments) do not ameliorate risks posed by decontextualized healthcare data [22,32–34]. Because these approaches intervene only after data have been produced, they cannot confront the contextual and structural inequalities embedded in healthcare data at its inception [3,35]. Addressing the challenges posed by decontextualized healthcare data requires reconceptualizing the entire AI lifecycle, from decisions about when and what data are collected, to model development, deployment, and external validation. They require stakeholder acknowledgement that data is fundamentally shaped by upstream social, political, and institutional forces rather than something to be manipulated with downstream technical considerations [3,35–38].

Meaningfully addressing decontextualized healthcare data requires genuine collaboration with communities and domain experts who understand the lived contexts of data generation. Indigenous Data Sovereignty principles demonstrate that when communities exercise authority over what data is collected, how it is interpreted, and for what purposes it is used, data completeness and fidelity improve because structural barriers to documentation are directly addressed [39–43]. Likewise, clinicians, nurses, social workers, allied healthcare workers and patient communities hold essential contextual expertise that enables assessment of whether data accurately capture clinical phenomena or instead reflects broader social inequities or contextual circumstance [3,11,35,44]. Their involvement must be foundational, not peripheral; to properly contextualize the data we use in healthcare.

Evaluation of model performance must also extend beyond statistical performance. The central question should not be “does this model perform well?” but “should this model exist *at all*, and under what conditions?” [37,38,45–47]. This necessitates participatory forms of validation that assess whether systems reinforce existing inequities or support community-defined goals [37–41,47–49].

Technical innovations in fairness, privacy, and distributed computational approaches that limit raw data extraction and secondary data use without local oversight should function as guardrails, not primary solutions, within a reimagined lifecycle where context precedes computation [22,45,46,50–55]. This paradigm shift requires institutional incentives that prioritize transparency, community involvement, community benefit, and contextual depth over technical novelty and model performance [37,38,47]. Only through such systemic transformation can AI systems avoid amplifying health inequities deeply rooted in the use of decontextualized healthcare data.

## 4. Conclusion

No amount of algorithmic refinement or sophisticated modeling techniques can compensate for datasets that fundamentally fail to capture the context of their own creation. Throughout this paper, we have examined the structural, epistemic, and algorithmic dimensions of decontextualized healthcare data, emphasizing that as AI becomes more deeply embedded in clinical decision-making, the risks of training models on such data grow increasingly urgent.

Rather than aiming for mathematical accuracy, we must foster a paradigm shift that moves toward deep, interdisciplinary collaboration. We must recognize missing, incomplete, and inaccurate data not as a statistical nuisance, but an ethical and systemic signal. This requires a concerted effort between the machine learning community and domain experts; clinicians, healthcare workers, and community representatives, who understand data context. It is only through this partnership that we can begin to build models that account for the complex realities and what data is trying to communicate.

Ultimately, we must reimagine AI systems not simply as tools of optimization, but as opportunities for repair, systems that do not obscure what is decontextualized, but illuminate it, giving voice to those historically left out, and shaping a more equitable and accountable future.

## References

[1] Pham TM, Pandis N, White IR. Missing data: issues, concepts, methods. Semin Orthod. 2024;30(1):37–44. doi: 10.1053/j.sodo.2024.01.007

[2] Emmanuel T, Maupong T, Mpoeleng D, Semong T, Mphago B, Tabona O. A survey on missing data in machine learning. J Big Data. 2021;8(1):140. doi: 10.1186/s40537-021-00516-9 34722113 PMC8549433

[3] Mitchell WG, Wawira JG, Celi LA. Rebooting artificial intelligence for health. PLOS Glob Public Health. 2025;5(1):e0004171. doi: 10.1371/journal.pgph.0004171 39823406 PMC11741560

[4] Twersky SE, Jefferson R, Garcia-Ortiz L, Williams E, Pina C. The impact of limited english proficiency on healthcare access and outcomes in the U.S.: a scoping review. Healthcare (Basel). 2024;12(3):364. doi: 10.3390/healthcare12030364 38338249 PMC10855368

[5] Mehta NK, Allam S, Mazimba S, Karim S. Racial, ethnic, and socioeconomic disparities in out-of-hospital cardiac arrest within the United States: now is the time for change. Heart Rhythm O2. 2022;3(6Part B):857–63. doi: 10.1016/j.hroo.2022.07.009 36588995 PMC9795269

[6] Mitchell WG, Deane A, Brown A, Bihari S, Wong H, Ramadoss R, et al. Long term outcomes for Aboriginal and Torres Strait Islander Australians after hospital intensive care. Med J Aust. 2020;213(1):16–21. doi: 10.5694/mja2.50649 32484925

[7] Greenwood BN, Carnahan S, Huang L. Patient-physician gender concordance and increased mortality among female heart attack patients. Proc Natl Acad Sci U S A. 2018;115(34):8569–74. doi: 10.1073/pnas.1800097115 30082406 PMC6112736

[8] Kyriacou PA, Charlton PH, Al-Halawani R, Shelley KH. Inaccuracy of pulse oximetry with dark skin pigmentation: clinical implications and need for improvement. Br J Anaesth. 2023;130(1):e33–6. doi: 10.1016/j.bja.2022.03.011 35430087

[9] Sjoding MW, Dickson RP, Iwashyna TJ, Gay SE, Valley TS. Racial bias in pulse oximetry measurement. N Engl J Med. 2020;383(25):2477–8. 33326721 10.1056/NEJMc2029240 PMC7808260

[10] Pantielieiev D. Garbage in, garbage out: how to stop your AI from hallucinating; 2025. https://shelf.io/blog/garbage-in-garbage-out-ai-implementation/

[11] Celi LA, Cellini J, Charpignon M-L, Dee EC, Dernoncourt F, Eber R, et al. Sources of bias in artificial intelligence that perpetuate healthcare disparities: a global review. PLOS Digit Health. 2022;1(3):e0000022. doi: 10.1371/journal.pdig.0000022 36812532 PMC9931338

[12] Trout MJ, Henson G, Senthuran S. Characteristics and outcomes of critically ill Aboriginal and/or Torres Strait Islander patients in North Queensland. Med J Austr. 2016. 10.1177/0310057X1504300212 25735688

[13] Stephens DP. Critical illness and its impact on the Aboriginal people of the top end of the Northern Territory, Australia. J Anaesth Intensive Care. 2003. 10.1177/0310057X0303100310 12879676

[14] McDonald SP, Russ GR. Burden of end-stage renal disease among indigenous peoples in Australia and New Zealand. Kidney Int Suppl. 2003;(83):S123-7. doi: 10.1046/j.1523-1755.63.s83.26.x 12864890

[15] Zhao CC, Wright J. Estimating chronic disease prevalence among the remote Aboriginal population of the Northern Territory. Aust N Zeal J Public Health. 2008. 10.1111/j.1753-6405.2008.00245.x 18782390

[16] The health & welfare of Australia’s Aboriginal & Torres Strait Islander people. Australian Government; 2015. Available from: https://www.aihw.gov.au/reports-data/health-welfare-overview/indigenous-health-welfare/overview

[17] Einsiedel LJ, van Iersel E, Macnamara R, Spelman T, Heffernan M, Bray L, et al. Self-discharge by adult Aboriginal patients at Alice Springs Hospital, Central Australia: insights from a prospective cohort study. Aust Health Rev. 2013;37(2):239–45. doi: 10.1071/AH11087 23257238

[18] Durey A, Thompson SC, Wood M. Time to bring down the twin towers in poor Aboriginal hospital care: addressing institutional racism and misunderstandings in communication. Intern Med J. 2012;42(1):17–22. doi: 10.1111/j.1445-5994.2011.02628.x 22032537

[19] Henry BR, Houston S, Mooney GH. Institutional racism in Australian healthcare: a plea for decency. Med J Aust. 2004;180(10):517–20. doi: 10.5694/j.1326-5377.2004.tb06056.x 15139829

[20] Lee S, Ahn K, Cha M. Community-level socioeconomic status and outcomes of patients with out-of-hospital cardiac arrest. A systematic review and meta analysis. Medicine. 2021;3:e24170. 10.1097/MD.0000000000024170 PMC7837968 33546033

[21] Nichol G, Thomas E, Callaway CW, Hedges J, Powell JL, Aufderheide TP, et al. Regional variation in out-of-hospital cardiac arrest incidence and outcome. JAMA. 2008;300(12):1423–31. doi: 10.1001/jama.300.12.1423 18812533 PMC3187919

[22] Obermeyer Z, Powers B, Vogeli C, Mullainathan S. Dissecting racial bias in an algorithm used to manage the health of populations. Science. 2019;366(6464):447–53. doi: 10.1126/science.aax2342 31649194

[23] Teotia K, Jia Y, Link Woite N, Celi LA, Matos J, Struja T. Variation in monitoring: glucose measurement in the ICU as a case study to preempt spurious correlations. J Biomed Inform. 2024;153:104643. doi: 10.1016/j.jbi.2024.104643 38621640 PMC11103268

[24] Oca SR, Navas A, Leiman E, Buckland DM. Effect of language interpretation modality on throughput and mortality for critical care patients: a retrospective observational study. J Am Coll Emerg Physicians Open. 2021;2(4):e12477. doi: 10.1002/emp2.12477 34263246 PMC8253091

[25] Duronjic A, Ku D, Chavan S, Bucci T, Taylor S, Pilcher D. The impact of language barriers & interpreters on critical care patient outcomes. J Crit Care. 2023;73:154182. doi: 10.1016/j.jcrc.2022.154182 36368174

[26] Jacobi J, Bircher N, Krinsley J, Agus M, Braithwaite SS, Deutschman C, et al. Guidelines for the use of an insulin infusion for the management of hyperglycemia in critically ill patients. Crit Care Med. 2012;40(12):3251–76. doi: 10.1097/CCM.0b013e3182653269 23164767

[27] Krinsley JS, Bruns DE, Boyd JC. The impact of measurement frequency on the domains of glycemic control in the critically ill—a Monte Carlo simulation. J Diabetes Sci Technol. 2015;9(2):237–45. doi: 10.1177/1932296814566507 25568143 PMC4604588

[28] Sreedharan R, Martini A, Das G, Aftab N, Khanna S, Ruetzler K. Clinical challenges of glycemic control in the intensive care unit: a narrative review. World J Clin Cases. 2022;10(31):11260–72. 36387820 10.12998/wjcc.v10.i31.11260 PMC9649548

[29] Falciglia M, Freyberg RW, Almenoff PL, D’Alessio DA, Render ML. Hyperglycemia-related mortality in critically ill patients varies with admission diagnosis. Crit Care Med. 2009;37(12):3001–9. doi: 10.1097/CCM.0b013e3181b083f7 19661802 PMC2905804

[30] Sekalala S, Chatikobo T. Colonialism in the new digital health agenda. BMJ Glob Health. 2024;9(2):e014131. doi: 10.1136/bmjgh-2023-014131 38413105 PMC10900325

[31] Gianfrancesco MA, Tamang S, Yazdany J, Schmajuk G. Potential biases in machine learning algorithms using electronic health record data. JAMA Intern Med. 2018;178(11):1544–7. doi: 10.1001/jamainternmed.2018.3763 30128552 PMC6347576

[32] Little R, Rubin D. Statistical analysis with missing data. 3 ed. Wiley; 2019.

[33] Buuren Sv. Flexible imputation of missing data. Advanced guide on multiple and flexible imputation strategies; 2012.

[34] Troyanskaya O, Cantor M, Sherlock G, Brown P, Hastie T, Tibshirani R, et al. Missing value estimation methods for DNA microarrays. Bioinformatics. 2001;17(6):520–5. doi: 10.1093/bioinformatics/17.6.520 11395428

[35] Sambasivan N, Kapania S, Highfill H, Akrong D, Paritosh P, Aroyo LM. “Everyone wants to do the model work, not the data work”: Data Cascades in High-Stakes AI. In: Proceedings of the 2021 CHI Conference on Human Factors in Computing Systems. 2021;1–15. 10.1145/3411764.3445518

[36] Kim Y, Finn M. Epistemologies of missing data: covid data builders and the production and maintenance of marginalized covid datasets. SPIR. 2023. doi: 10.5210/spir.v2023i0.13438

[37] Ferryman K. The dangers of data colonialism in precision public health. Global Policy. 2021;12(S6):90–2. doi: 10.1111/1758-5899.12953

[38] Looi MK. What should decolonisation of medical institutions look like?. BMJ. 2023:2257. 37844936 10.1136/bmj.p2257

[39] Leonard K, Russo S, Martinez A, McElroy L. Our common agenda global digital compact March 2023: CARE statement for indigenous data sovereignty; 2023.

[40] McBride Kelly L, Wong D, Timothy A. Measuring what counts in Aboriginal and Torres Strait Islander care: a review of general practice datasets available for assessing chronic disease care. Aust J Prim Health. 2024;30:PY24017. doi: 10.1071/PY24017 38981000

[41] Geck MS, Cristians S, Berger-González M, Casu L, Heinrich M, Leonti M. Traditional herbal medicine in mesoamerica: toward its evidence base for improving universal health coverage. Front Pharmacol. 2020;11. 10.3389/fphar.2020.01160 PMC7411306 32848768

[42] Gray M, Williams K, Henderson R, Oster R, Samara W, Grant B, et al. Efforts to introduce an indigenous identifier in a Canadian Provincial Health Authority. IJIH. 2024;20(1). doi: 10.32799/ijih.v20i1.42171

[43] ONIX. Digital transformation made worry-free and reliable with automated data validation. ONIX; 2025. Available from: https://www.onixnet.com/blog/digital-transformation-made-worry-free-and-reliable-with-automated-data-validation/

[44] Lindahl C, Wagner S, Uldbjerg N, Schlütter JM, Bertelsen O, Sandager P. Effects of context-aware patient guidance on blood pressure self-measurement adherence levels. Health Informatics J. 2019;25(2):417–28. doi: 10.1177/1460458217717073 28701078

[45] Liu X, Rivera SC, Moher D, Calvert MJ, Denniston AK, SPIRIT-AI and CONSORT-AI Working Group. Reporting guidelines for clinical trial reports for interventions involving artificial intelligence: the CONSORT-AI Extension. BMJ. 2020;370:m3164. doi: 10.1136/bmj.m3164 32909959 PMC7490784

[46] World Health Organisation. Module 1: framework and metrics; 2017.

[47] The Lancet Digital Health. Decolonising health data. Lancet Digit Health. 2023;5(8):e477. doi: 10.1016/S2589-7500(23)00132-2 37507194

[48] Shaw J, Sekalala S. Health data justice: building new norms for health data governance. NPJ Digit Med. 2023;6(1):30. doi: 10.1038/s41746-023-00780-4 36854964 PMC9972302

[49] Wylie L, McConkey S. Insiders’ insight: discrimination against indigenous peoples through the eyes of health care professionals. J Racial Ethn Health Disparities. 2019;6(1):37–45. doi: 10.1007/s40615-018-0495-9 29736617 PMC6347580

[50] Zhang B, Lemoine B, Mitchell M. Mitigating unwanted biases with adversarial learning. arXiv pre-print server; 2018.

[51] Norori N, Hu Q, Aellen FM, Faraci FD, Tzovara A. Addressing bias in big data and AI for health care: a call for open science. Patterns (N Y). 2021;2(10):100347. doi: 10.1016/j.patter.2021.100347 34693373 PMC8515002

[52] Mitchell M, Wu S, Zaldivar A, Barnes P, Vasserman L, Hutchinson B. Model cards for model reporting. ACM; 2019.

[53] Silva PJ, Rahimzadeh V, Powell R, Husain J, Grossman S, Hansen A, et al. Health equity innovation in precision medicine: data stewardship and agency to expand representation in clinicogenomics. Health Res Policy Syst. 2024;22(1):170. doi: 10.1186/s12961-024-01258-9 39695714 PMC11657299

[54] Rossi N, Golinelli L, Bersani F, Geraci M. A retrospective analysis of the factors associated with surgical checklist compliance using data from a local health unit in Italy, 2018-2021. J Eval Clin Pract. 2023;29(8):1372–9. doi: 10.1111/jep.13912 37525361

[55] Abdallah M, Hammad A, Staegemann D. A data collection quality model for big data systems. In: IEEE, editor. 2023 International Conference on Information Technology (ICIT). Jordan: IEEE; 2023.

